# What If We Let It Fibrillate? New Perspective on Atrial Fibrillation in Sepsis

**DOI:** 10.1007/s11596-026-00185-w

**Published:** 2026-03-23

**Authors:** Matteo Guarino, Roberto De Giorgio

**Affiliations:** 1 Department of Translational Medicine, St. Anna University Hospital of Ferrara, Ferrara, 44124 Italy; 2 Emergency Department, S. Anna University Hospital of Ferrara, Ferrara, 44124 Italy

**Keywords:** Atrial fibrillation, Arrhythmias, Emergency medicine, Sepsis, Septic shock

## Abstract

Atrial fibrillation (AF) is a frequent arrhythmia in sepsis patients and is traditionally regarded as a harmful epiphenomenon requiring immediate correction. Its abrupt onset and association with adverse outcomes have driven clinicians to pursue aggressive rhythm or rate control strategies, which are often extrapolated from cardiology rather than sepsis-specific evidence. However, emerging data challenge this reflexive approach. Studies with ultrashort-acting beta-blockers, such as esmolol and landiolol, have shown that hemodynamic stabilization may occur even in the absence of sinus rhythm restoration. This raises a provocative question: could AF during sepsis represent not a pathological disturbance but rather a transient and potentially tolerable manifestation of sepsis-related cardiovascular stress? We propose the concept of “permissive AF,” shifting the focus from rhythm restoration to hemodynamic optimization and organ perfusion. Thus, AF is not framed as a physiological adaptation per se but as a maladaptive manifestation of sepsis-related cardiac injury that may be clinically tolerated, paralleling permissive strategies in critical care, such as permissive hypercapnia or restrictive transfusion thresholds. This does not imply therapeutic inaction; rather, it is a goal-directed approach that tolerates arrhythmia when adequate rate control and perfusion are achieved. This paradigm challenges established clinical instincts and underscores the need for individualized management. Recognizing AF as a potential adaptive rhythm reframes research priorities: identifying biomarkers of atrial inflammation, stratifying patients according to hemodynamic impact, and testing permissive versus corrective strategies in prospective trials. The permissive AF strategy proposed herein is expected to foster patient-centered care and reduce iatrogenic harm in the complex interplay between sepsis and the heart.

**Graphical Abstract:**

Clinical dilemma of atrial fibrillation during sepsis. Electrical cardioversion is depicted with traditional defibrillator pads, symbolizing rhythm-focused intervention (left). Pharmacologic rate control is represented by beta-blockers and a perfused heart, highlighting a patient-centered approach on the basis of hemodynamic stability (right).

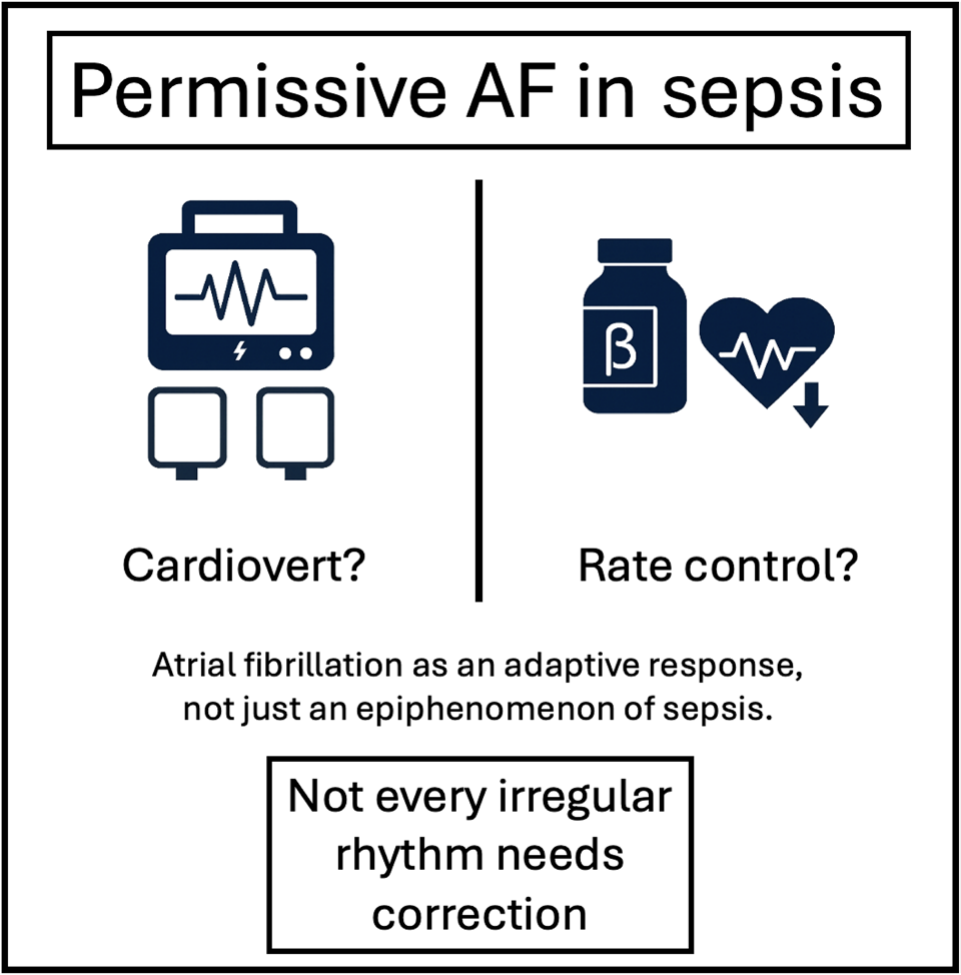

## Introduction

Atrial fibrillation (AF) is one of the most common arrhythmias encountered in critically ill patients, particularly those with sepsis, where its incidence ranges from 10% to 20% and is even greater in patients with septic shock or advanced age and preexisting cardiac disease [1, 2]. Its sudden appearance during an already precarious clinical course often elicits a reflexive, almost instinctive response: restoring sinus rhythm at all costs. This impulse is grounded in decades of cardiology teaching, in which AF is framed as a pathologic disturbance, a marker of instability, and a predictor of worse outcomes. Indeed, new-onset AF during sepsis has been consistently associated with increased mortality, prolonged intensive care unit (ICU) stay, and increased risk of multiorgan failure [1, 2]. The clinical narrative that follows is therefore straightforward: AF is bad, and we must correct it.

However, what if this story is incomplete? What if AF in sepsis is not merely a maladaptive epiphenomenon but probably a tolerable manifestation of stress or a transient response of the inflamed heart under systemic stress? This question lies at the core of how we conceptualize cardiovascular physiology in critical illness. Thus, we must reconsider whether rhythm restoration is inherently beneficial or whether the arrhythmia itself might serve as a compensatory mechanism in the face of a cytokine storm, autonomic dysregulation, and profound microcirculatory dysfunction [3, 4].

Current management strategies (whether rhythm control with amiodarone or cardioversion or rate control with beta-blockers and calcium channel blockers) are largely extrapolated from nonseptic populations [2, 5]. However, their efficacy in this unique inflammatory milieu is limited. Rhythm control often fails during the acute phase, where atrial tissue and conduction pathways are deeply altered by cytokine-mediated injury and excess catecholamines. Rate control, while conceptually attractive, is fraught with hemodynamic risk in patients dependent on vasopressors or struggling with borderline perfusion [2, 5]. Behind these interventions lies an unquestioned assumption: is sinus rhythm always better?

This manuscript explores an alternative paradigm, i.e., proposing the concept (or strategy) of “permissive AF” in sepsis. Rather than fighting arrhythmia, this approach tolerates it when adequate perfusion and rate control are achieved, prioritizing hemodynamic stability over electrical order. The concept mirrors other critical care strategies where traditional “normal” values are intentionally abandoned, permissive hypercapnia to protect the lungs in acute respiratory distress syndrome (ARDS) [6], permissive hypoxemia to avoid oxygen toxicity [7], and restrictive transfusion thresholds to minimize iatrogenesis [8]. Could AF in sepsis be another physiologic deviation that physicians may accept?

## Pathophysiological Rationale: The Inflamed Heart and Atrial Fibrillation

Sepsis profoundly affects the cardiovascular system, extending beyond the well-known depression of myocardial contractility, which includes complex electrophysiological disturbances. The atria, with their thin walls, abundant autonomic innervation, and high metabolic demand, are particularly vulnerable to this systemic insult. Several overlapping mechanisms create a fertile substrate for AF.

First, the cytokine storm characteristic of sepsis induces widespread myocardial inflammation. Elevated levels of interleukin-6, tumor necrosis factor-alpha, and other mediators disrupt calcium handling, alter ion channel expression, and promote electrical heterogeneity across the atrial myocardium [3, 4]. This biochemical remodeling lowers the threshold for reentry circuits and ectopic firing. Simultaneously, nitric oxide and reactive oxygen species contribute to oxidative stress, impairing gap junction function and further destabilizing conduction.

Second, the autonomic nervous system, often in a state of hyperadrenergic overdrive early in sepsis, produces profound sympathovagal imbalance. High levels of circulating catecholamines (both endogenous and exogenous from vasopressor therapy) increase automaticity and shorten atrial refractory periods [3]. Later, vagal surges and a fluctuating tone create a dynamic and unstable electrophysiologic milieu. This “autonomic storm” mirrors the erratic rhythm that emerges on the electrocardiogram (ECG).

Third, the microcirculatory dysfunction and capillary leakage inherent to sepsis promote myocardial edema and regional ischemia. The atria, particularly the thin-walled left atrium, are sensitive to these microstructural changes, which can impair coordinated contraction and trigger disorganized electrical activity. All these factors converge to create AF not only as an incidental byproduct but also as a natural response to an organ under duress.

Seen through this lens, AF may be viewed as a transient, clinically tolerable manifestation of injury. In the setting of variable preload, fluctuating afterload, and dynamic autonomic input, rapid irregular atrial activation could represent the heart’s attempt to maintain forward flow when synchronized contraction is temporarily compromised. This concept aligns with observations that AF often resolves spontaneously as sepsis abates, suggesting that it is not a fixed arrhythmia but rather a state-dependent rhythm reflecting the inflammatory environment rather than an irreversible structural disease [9].

## Therapeutic Implications: From Rhythm Obsession to Hemodynamic Priorities

Traditional management strategies for new-onset AF in the ICU reflect the cardiology dogma: restoring sinus rhythm or at least slowing the ventricular response. In sepsis, these approaches face unique challenges. Electrical cardioversion is notoriously ineffective during the acute inflammatory phase; recurrence rates are high, and procedural sedation carries risks in unstable patients [2, 5]. Amiodarone, the most commonly used antiarrhythmic agent, often fails to maintain sinus rhythm and is associated with hypotension, QT prolongation, and drug interactions. Calcium channel blockers are typically avoided in shock due to negative inotropic effects. While electrical cardioversion remains a Class I indication for AF with immediate hemodynamic collapse, its efficacy is often short-lived in the presence of ongoing systemic inflammation [5]. With respect to pharmacological options, digoxin may be considered for rate control, although its slow onset and renal clearance limit its utility in acute septic shock. In specific regions, pure potassium channel blockers, e.g., nifekalant, have been explored for rhythm control, although their role in the context of septic cytokine storms remains to be fully elucidated [5, 10].

Beta-blockers, which have long been considered contraindicated in hypotensive patients, have re-emerged as potential game changers. Studies with ultrashort-acting agents (esmolol and landiolol) have shifted the paradigm. In septic shock patients with persistent tachyarrhythmia, esmolol infusion has improved stroke volume, arterial elastance, and even signals of survival benefit despite patients remaining in AF [11]. Owing to its high beta-1 selectivity and extremely short half-life, landiolol has shown promise in small, randomized trials, offering precise rate control with minimal impact on blood pressure [12–14].

The striking element of these studies is that hemodynamic improvement occurred without rhythm conversion. Patients continued to fibrillate, yet perfusion markers, lactate clearance, and oxygen delivery improved. Decoupling the hemodynamic benefit from sinus rhythm restoration suggests that the therapeutic target may not be the rhythm itself but rather the heart rate, diastolic filling, and autonomic modulation. However, it should be acknowledged that high-quality evidence derived by comparing rate versus rhythm control in sepsis patients is currently lacking. Most available data are extrapolated from nonseptic populations or focus on rate control alone. Furthermore, new-onset AF in sepsis often serves as a marker of disease severity and is frequently triggered by the interventions needed to maintain perfusion, such as aggressive fluid resuscitation and catecholamine support [4, 5]. This is the foundation of a “permissive AF” approach. Rather than an aggressive push for electrical normality, clinicians focus on achieving a ventricular rate compatible with adequate cardiac output and tissue perfusion. Practical goals might include a heart rate <110 bpm, stabilization of the mean arterial pressure, and objective evidence of tissue oxygenation. Specifically, monitoring dynamic parameters, such as central venous oxygen saturation (ScvO_2_) and the veno-arterial PCO_2_ difference (ΔPCO_2_), provides critical information [15, 16]. Indeed, a stable or improved ScvO_2_ and a ΔPCO_2_ < 6 mmHg suggest that oxygen delivery is adequate despite arrhythmia [17]. Lactate trends and urine output become as important as those of the ECG. Rhythm control is not abandoned but is reserved for those patients in whom AF clearly precipitates hemodynamic collapse or coexists with severe structural disease.

Positioning this strategy within a broader critical care framework highlights its logic. Just as we accept permissive hypercapnia to prioritize lung protection in ARDS [6] and tolerate lower oxygen saturation to avoid oxygen toxicity [7], accepting AF in the septic heart prioritizes function over form.

## The Role of Bedside Echocardiography: Tailoring a Permissive AF Strategy

Implementing a “permissive AF” approach requires more than simply tolerating an irregular rhythm; it demands continuous reassessment of the heart’s ability to sustain adequate output under inflammatory stress. Bedside echocardiography provides a unique window into this dynamic physiology, guiding physicians beyond heart rate targets to evaluate actual perfusion and cardiac functions.

In septic patients with AF, echocardiography can be used to clarify whether the arrhythmia is hemodynamically tolerated or whether it is precipitating instability. Assessment of atrial size, ventricular filling pressures, and diastolic function can help identify patients whose cardiac reserve is too limited to accommodate the loss of atrial contraction. Conversely, a hyperdynamic ventricle with preserved stroke volume despite irregular filling may indicate that the rhythm can be safely accepted while focusing on rate control and perfusion.

Equally important is the integration of fluid responsiveness and fluid tolerance into the decision-making process. As highlighted in sepsis management frameworks [18], dynamic ultrasound indices, such as the left ventricular outflow tract velocity‒time integral (VTI), inferior vena cava variability, or passive leg-raising response, can be used to determine whether tachyarrhythmia reflects residual preload dependency or whether further fluids would only worsen congestion. AF itself often blunts traditional preload markers, making echocardiographic guidance critical to avoid both underresuscitation and fluid overload. Bedside imaging also allows real-time evaluation of therapeutic effects. Rate control with beta-blockers, particularly esmolol or landiolol, can be titrated while monitoring VTI, stroke volume, and indices of diastolic filling. This transforms “permissive AF” from a theoretical position into a dynamic, goal-directed strategy: the arrhythmia is not ignored but is actively monitored in the context of systemic perfusion. In this context, echocardiography has become the operational arm of the permissive AF concept. It bridges the gap between electrical rhythm and functional adequacy, enabling physicians to distinguish AF as a tolerable manifestation of sepsis-related stress from AF as a driver of hemodynamic failure. Integrating ultrasound into this paradigm reinforces rhythm-centric thinking versus individualized, physiology-based care [3, 11, 19].

## Clinical Mindset Shift: Learning to Tolerate the Abnormal

The greatest limit to permissive AF is likely not physiological but rather a psychological barrier. The irregular rhythm triggers a deep-seated clinical reflex: something that is wrong should be fixed. Decades of training reinforce sinus rhythm as synonymous with stability. Challenging that paradigm requires a deliberate shift in mindset, such as the one critical care has undergone in other domains.

Permissive hypercapnia was once heresy; allowing CO_2_ to rise was perceived as abandoning the patient. In contrast, evidence has demonstrated that rigid normalization of PaCO_2_ causes more harm via ventilator-induced lung injury [6]. The acceptance of hypoxemia to avoid oxygen toxicity has gone against every instinct to “correct numbers” but has become standard in ARDS [7]. Similarly, restrictive transfusion strategies challenge the dogma of hemoglobin normalization and ultimately reduce mortality and complications [8].

Permissive AF follows the same ideal pathway: questioning whether our drive to correct reflects physiology or bias. It does not advocate passivity. Conversely, it demands active, goal-directed care: frequent reassessment of perfusion, judicious use of rate control agents, and clear criteria as to when rhythm control is necessary. It also emphasizes heterogeneity: not all septic AFs are adaptive. Patients with severe diastolic dysfunction, ischemia, or limited cardiac reserve may not tolerate even well-controlled AF. Identifying these subgroups is of critical clinical relevance.

Future research should address these questions. Prospective trials comparing permissive versus corrective strategies could clarify outcomes. Biomarkers of atrial inflammation, autonomic tone, or myocardial strain may help stratify patients. Bedside tools that integrate echocardiography, rate control targets, and perfusion metrics could guide real-time decisions. More broadly, this mindset shift reframes AF in sepsis from an enemy to be suppressed into a signal to be interpreted. It invites clinicians to listen to the irregular rhythm not as chaos but as communication from the inflamed heart, asking not for immediate correction but for understanding and support.

## Thromboembolic Considerations in Permissive AF

A significant challenge in tolerating AF is the inherent risk of systemic embolism, which is further amplified by the prothrombotic milieu of sepsis. While the permissive approach prioritizes hemodynamic stability and avoids iatrogenic harm, physicians should carefully evaluate the indication for anticoagulation on an individual basis. Conventional risk scores, such as CHA_2_DS_2_-VA, may provide a general framework, although their applicability in critically ill septic patients is limited, as both thrombotic and bleeding risks are dynamically influenced by inflammation, endothelial dysfunction, coagulopathy, and invasive procedures [4, 20, 21].

In the hyperacute phase of sepsis, full-dose anticoagulation is frequently precluded by high bleeding risk, thrombocytopenia, or ongoing organ support. In such settings, short-acting agents, delayed initiation strategies, or mechanical prophylaxis may represent the only feasible options [18]. The permissive AF paradigm does not imply neglecting thromboembolic risk; rather, anticoagulation decisions should be integrated into a broader, patient-centered assessment that evolves with clinical stabilization [4, 20].

## Conclusion

AF during sepsis has long been considered a malignant rhythm that demands immediate suppression. However, mounting evidence suggests a more accurate approach. AF may, in some patients, represent not a failure but a transient, adaptive response to overwhelming inflammatory stress. Embracing a strategy of “permissive AF” shifts the therapeutic strategy from electrical order to functional adequacy and from reflexive rhythm control to patient-centered hemodynamic optimization. This paradigm does not discard traditional interventions but contextualizes them, integrating rate control, perfusion targets, and individualized thresholds. It also invites a broader reflection: in the complex pathophysiology of sepsis, not every deviation from “normal” warrants immediate correction. Survival sometimes relies on irregular beats.

Prospective trials comparing permissive and corrective approaches, along with mechanistic studies of atrial inflammation, will be essential to refine this hypothesis. Until then, physicians are challenged to pause before shock, to listen to the disordered rhythm of the septic heart, and to ask: what if we let it fibrillate?

## Data Availability

Not applicable.
